# Climate futures: Scientists' discourses on collapse versus transformation

**DOI:** 10.1111/bjso.12840

**Published:** 2024-12-14

**Authors:** Samuel Finnerty, Jared Piazza, Mark Levine

**Affiliations:** ^1^ Department of Psychology Lancaster University Lancaster UK

**Keywords:** activism, climate change, environmental discourse, future, identity, science, temporality, uncertainty

## Abstract

The climate and ecological crisis poses an unprecedented challenge, with scientists playing a critical role in how society understands and responds. This study examined how 27 environmentally concerned scientists from 11 countries construct the future in the context of climate change, applying a critical discursive psychology analysis. The degree to which the future is constructed as predetermined or transformable impacts both the urgency and scope of proposed actions. Along a temporal spectrum from fixed and inevitable to contingent and transformable, scientists drew upon shared discourses of social and ecological collapse. The degree of fixity or openness in scientists' talk about the future shaped the range of arguments available, demonstrating varying levels of argumentative flexibility when framing solutions to climate change. At the fixed end, the future was presented as beyond human intervention, echoing doomist discourse. By contrast, more open framings presented collapse not as inevitable but as transformable through human agency. Here, collapse discourses were presented as warnings, motivating arguments that drew upon a wide array of strategies from collective action to technological innovation. These constructions of the future highlight scientists' role in shaping societal discourse and framing what actions are seen as viable or necessary to address the climate crisis.

## INTRODUCTION

The scientific community's discourse on the future is critical in shaping humanity's response to climate change (IPCC, 2023) and biodiversity loss (IPBES, 2019). As trusted actors (Cologna, Mede, et al., 2024), scientists have long been catalysts for driving societal progress and enhancing human welfare (Independent Group of Scientists appointed by the Secretary‐General, 2019; IPCC, 2022; World Health Organization, 2023). However, the narrative of scientific progress, which has long promised a future of prosperity (Pinker, 2018; Porter, 2003), is challenged by this crisis. The environmental crisis introduces profound uncertainties (UNDP, 2022; World Health Organization, 2023). Within this context, there are a diversity of future perspectives within the scientific community. Optimism persists among some, arguing that scientific ingenuity can overcome these environmental hurdles (O'Neill, 2023; Ritchie, 2024). Others signal a time of planetary uncertainty and probable decline (Bradshaw et al., 2021). Recognizing the urgency of these issues, a growing number of scientists are stepping beyond research roles to advocate for policy changes and participate in social movements, leveraging their expertise to guide humanity towards a more sustainable future (Capstick et al., 2022; Gardner et al., 2021; Oza, 2023). Although this group represents a minority within the broader scientific community, a global survey of over 9000 researchers found that 23% of scientists report engaging in legal protests and 10% have participated in civil disobedience (Dablander et al., 2024). While these figures provide valuable insights, this may overrepresent scientists who are particularly concerned about climate change, suggesting the numbers may not fully reflect activism levels across the entire scientific community. Nevertheless, this is the most comprehensive data available on this issue to date.

Critical discursive psychology (CDP) (Edley & Wetherell, 2001; Wetherell, 1998) may provide a powerful framework for examining how scientists construct and negotiate the future. CDP as a method (Edley & Wetherell, 2001) enables analysis both of how talk reflects wider discourses and how participants employ these discourses in conversation, and position themselves in relation to them, for specific rhetorical purposes (Billig, 1987; Billig et al., 1988). Our research team recently used CDP to explore how scientists navigate the ‘ideological dilemma’ (Billig et al., 1988) between maintaining scientific objectivity and engaging in activism (Finnerty et al., 2024a). Set against the background of scientists' increasing advocacy and engagement with social movements (Dablander et al., ; Finnerty et al., 2024b), the current study builds on this initial work to explore how scientists' discussions about the future are constructed and mobilized to support specific pathways for climate action.

### Theoretical background and context

#### World‐making, social movements, and the future

Collective beliefs about what the world is, what it could be, and what it should be, profoundly influence the worlds we create (Power et al., 2023; Taylor, 2003). These shared visions, or social imaginaries—a shared understanding of social existence—inform expectations and guide our actions (Taylor, 2003). Climate change increases uncertainty about the future (IPCC, 2023; UNDP, 2022), fostering competing visions within climate change discourse that influence mitigation responses (Stoddard et al., 2021). Critics underscore how prevailing discourses and imaginaries regarding *technological optimism, business‐as‐usual*, and *doomism* have failed to significantly reduce global emissions or inspire meaningful climate action (Celermajer et al., 2024; Lamb et al., 2020; Stoddard et al., 2021). Technological optimism places faith in future emissions reduction technology (Lamb et al., 2020; Peeters et al., 2016) and business‐as‐usual assumes current practices are sufficient to adequately address climate change (Celermajer et al., 2024; Thierry et al., 2023). Conversely, the doomist imaginary adopts a fatalistic outlook, asserting the futility of action (Celermajer et al., 2024). By constraining how people imagine the future, these perspectives narrow what responses seem feasible or possible. Amidst these discursive constraints, global environmental social movements which reject both status quo and fatalist visions have emerged. Groups like Extinction Rebellion and Fridays for Future mobilize around dire predictions for the future but advocate for collective, just, and equitable solutions to address these crises (Berglund & Schmidt, 2020; Extinction Rebellion, 2024; Fridays for Future, 2024), predicated on a form of ‘radical hope’ (Stuart, 2020).

#### Scientists' responses to the environmental crisis

Since 2017, some scientists have taken a more active role in environmental social movements, transitioning from research to direct action (Gardner & Wordley, 2019; Reardon et al., 2017). As part of groups like Scientists for Extinction Rebellion (S4XR, 2023) and (SR, 2023), actions include obstructing fossil fuel infrastructure (Oza, 2023), leaking the IPCC report (Scientist Rebellion, 2021), and pasting scientific papers to government buildings (Gardner et al., 2022; Gayle, 2022). These bold steps, sometimes leading to arrests (Gayle, 2022; Oza, 2023), reflect their commitment to environmental causes. Competing conceptions of how scientists should conduct themselves in relation to the climate crisis presents a dilemma for environmentally concerned scientists (Finnerty et al., 2024a). This dilemma lies between upholding traditional scientific values and addressing the urgency of these crises. The traditional conception of the scientist—as impartial and objective, and discouraged from political action (Betz, 2013; Castree, 2019; Lackey, 2007; Nelson & Vucetich, 2009; Nielsen, 2001; Sedlak, 2016)—is challenged by critics who argue that this separation is morally and intellectually unsound given the scale of the problem (Capstick et al., 2022; Reardon et al., 2017; Rodgers, 2023). These tensions are reflected in diverse scientist identity constructions which can either support or hinder action. Survey research indicates that scientists who have reconciled the values of science with activism, and perceive a moral duty to act, are more likely to engage in activism (Finnerty et al., 2024b). This group of scientists is also less likely to support techno‐solutionist approaches, which often emphasize future technological fixes over systemic change^1^ (Finnerty et al., 2024b). Further interview research identified varied discursive strategies scientists use to frame and manage the ‘ideological dilemma’ (Billig et al., 1988) between upholding traditional scientific values and addressing the urgency of these crises (Finnerty et al., 2024a). One unexplored aspect of scientists' talk involves scientists' constructions of the future, an area ripe for exploration as the climate crisis fundamentally involves anticipating and mitigating future impacts.

### The current research

Interviews with 27 environmentally concerned scientists were conducted to understand their views on the climate and biodiversity crises and their actions in response. Initial analysis detailed varied scientist identity constructions and how these are used rhetorically to manage the dilemma of the scientist‐activist (Finnerty et al., 2024a). Prompted by the ‘Futures Focussed Social Psychology’ BJSP open call, a second reading of these interviews focused on a different aspect of this discourse: how scientists construct and mobilize visions of the future. Humans are inherently future‐oriented, with thoughts about the future often involving ‘preparation for action to bring about desired outcomes’ (Baumeister et al., 2016). Yet, the uncertainties surrounding environmental‐change trajectories and the most effective mitigation strategies (Biermann et al., 2022; Capstick et al., 2022; Castree, 2019; Chenoweth & Stephan, 2011; Hamilton, 2013; IPCC, 2023; Irvine et al., 2019; Mathew, 2022; Ritchie, 2024; Smith et al., 2023; Winkelmann et al., 2022) pose critical dilemmas for scientists: which vision of the future to accept, how to tackle the challenges presented, and which solutions to trust? Here, we examine how scientists draw on available climate discourse to manage these uncertainties and construct the future through talk, and how these constructions are used to justify and critique responses to climate change. This analysis draws on CDP (Edley & Wetherell, 2001; Wetherell, 1998), which may be used to examine how talk reflects wider discourses and is employed rhetorically to construct meaning and navigate social dilemmas.

## METHOD

The interviews were preregistered (see here for preregistration). Participants, recruited from a survey on their views on the climate and biodiversity crisis and their actions (Finnerty et al., 2024b), provided informed consent and were not compensated. To protect participant confidentiality, the interviews are not publicly available, but analysis enquiries can be directed to the authors. Ethical approval was granted by the Lancaster University Faculty of Science and Technology to conduct interviewees with scientists on their responses to the environmental crisis. The pre‐registration details a planned thematic analysis. However, as data analysis progressed, we recognized that CDP (Edley & Wetherell, 2001; Wetherell, 1998) would offer insights into the rhetorical and social functions of scientists' talk, particularly given the focus on how they construct and mobilize visions of the future. Thus, while the initial tagging and coding of text overlapped with thematic analysis, we transitioned to CDP for a richer exploration of how language shapes and reflects broader discourses on climate change.

### Participants

Twenty‐seven natural and social scientists were interviewed (59% Male, *M*_*age*_ = 40.19 years, *SD* = 12.93, range = 24–77). See Table 1 for sample description. Participants were recruited via an advert in a survey on scientist activism (see Supplements for recruitment strategy). Participants were identified by a number, their gender, and discipline. Interviews were conducted from June 2022 to December 2022. The international makeup of the sample reflects the global nature of the scientific community and the non‐profit organizations organizing scientists' responses to the climate crisis. However, we acknowledge that the UK is overrepresented in our sample, reflecting the UK's prominent role in recent climate activism and the establishment of key global activist groups such as Extinction Rebellion (of which Scientists for Extinction Rebellion are a part) and Scientist Rebellion. It is important to note that the sample includes a higher‐than‐usual proportion of participants engaging in direct action (37%), compared to a recent larger survey of over 9000 scientists, where 10% reported direct action (Dablander et al.,). This activist skew reflects our recruitment strategy and the focus on environmentally concerned scientists but may not fully represent the broader scientific community.

**TABLE 1:** Sample description.

Number of participants	27
Gender distribution	Male: 59% 41% Female
Age (Years)	*Mean =* 40.19, *SD =* 12.93, Range: 24–77
Nationality	UK: 44.44%, USA: 11.11%, Australia: 7.4%, Belgium: 7.4%, Ireland: 7.4%, Estonia: 3.7%, Germany: 3.7%, India: 3.7%, Malta: 3.7%, South Africa: 3.7%, Spain: 3.7%
Country of residence	UK: 74.07%, Australia: 11.11%, Ireland: 7.4%, Austria: 3.7%, Spain: 3.7%
Educational attainment	PhD: 59.26%, Masters: 37.04%, Bachelors: 3.7%
Political ideology (economic)	*Mean =* 1.58, *SD* = 0.64 (Scale: 1 = Very liberal, 4 = Moderate, 7 = Very conservative)
Political ideology (social)	*Mean =* 1.46, *SD =* 0.65 (Scale: 1 = Very liberal, 4 = Moderate, 7 = Very conservative)
Type of science	59.26% (16) Natural Scientists and 40.74% (11) Social Scientists
Fields of academic study	Agricultural Sciences, Anthropology, Biology, Chemistry, Computer Science, Earth Science, Ecology, Engineering & Technology, Environmental Monitoring, Geography, Mathematics, Medical and Health Sciences, Psychology, Physics, Sociology
Membership in direct‐action groups	48.14% were part of direct‐action activist groups such as Scientists for Extinction Rebellion, Scientist Rebellion, Just Stop Oil, and Extinction Rebellion
Engagement in arrestable action	37.03% (10) had taken direct action, with 22.22% (6) participants having been arrested for their actions at least once
Membership in scientist‐led groups	33.33% (9) were members of direct‐action groups that used the scientist identity as part of their actions, such as Scientists for Extinction Rebellion and Scientist Rebellion.

### Interviews

Semi‐structured interviews were conducted online via Microsoft Teams by the first author. All interviews were conducted in English as all interviewees were fluent English speakers. They investigated scientists' views of the climate and ecological crisis, their actions, and views on activism (Table S1). Two questions towards the end prompted participants to discuss the future. Additionally, given the interview context, discussions about the future naturally emerged throughout conversation. The schedule served as a topic guide rather than a prescriptive set of questions, allowing for flexibility and adaptation to each interviewee. The opening question was used to orient to each interviewee, and questions were adapted as required ensuring topics were broadly covered.

The first author did not disclose his activism unless specifically asked, to ensure that participants felt comfortable expressing their genuine thoughts and opinions about activism. Where applicable, disclosure did stimulate insightful conversations about the role of activism in research.

### Reflexivity statement

We recognize that, as researchers, we are not detached observers (Geertz, 1973), but active participants in environmental discourse. Throughout this study, we reflected on our own positionality as researchers to enhance the credibility and depth of our analysis. As environmentally concerned psychologists, we grapple with questions about our commitments and actions, much like our interviewees. Within our team, there is no consensus on which actions are most effective or appropriate, nor on how to reconcile these with our academic roles. This diversity among us mirrors the varied interviewee perspectives, helping us to understand the ideological dilemmas faced by scientists. Likewise, there is no consensus among us regarding what the future looks like, nor which actions should be taken to shape it. These diverse perspectives allowed us to approach the study without preference towards any specific future scenario or action strategy. Nevertheless, our motivation to conduct this research is driven by a belief in its importance. Understanding how scientists navigate the future is vital for fostering informed discussions within the scientific community about responses to the climate crisis. To manage the potential influence of our perspectives, we acknowledged our motivations while striving to maintain methodological rigour and impartiality to accurately represent participants' views.

### Analytic approach

We examined scientists' future constructions using a CDP approach. CDP attends to how language constructs identity, negotiates power relations, and challenges or reproduces dominant discourses and ideologies (Tileagă & Stokoe, 2015; Wetherell, 1998), acknowledging individuals' dual role as shaped by and shapers of discourse (Edley, 2001; Potter & Wetherell, 1987). Our analysis focused on *interpretive repertoires*—culturally shared ways of talking about and understanding the world—and how these are employed in discussions about the future (Wetherell, 1998). CDP emphasizes the rhetorical nature of thinking, with speakers using language to achieve specific aims (Billig et al., 1988). For instance, scientists may use language to argue for the necessity of direct action to avoid undesirable futures (Capstick et al., 2022). This makes it essential to examine the *subject positions* scientists adopt, which are the relational and social locations constructed for themselves or others to position themselves within the broader discourse (Edley, 2001; Wetherell, 1998). Our analysis paid close attention to metaphors, framing, and persuasive strategies.

All analysis was completed by the authors following standardized steps for discourse analysis (Potter & Wetherell, 1987). Analysis of the future‐related interview content followed a previous analysis detailing how scientists balance traditional scientific values with the urgency of the climate and ecological crisis (Finnerty et al., 2024a). Analysis of future‐related material involved several rounds of reading and coding the interview material, focusing on how talk about the future was constructed and used. We coded future‐related material and examined how scientists' talk reflected broader social discourses and served as a form of social action (Edley & Wetherell, 2001) and performed specific rhetorical functions (Billig, 1987).

Given the uncertainties surrounding climate change and its potential impacts, we applied an ‘ideological dilemmas’ lens (Billig et al., 1988) which facilitated an exploration of the conflicting imperatives and imagined futures within scientific and environmental discourses. This highlighted how scientists engaged with contrasting futures in the wider discourse (e.g. *dystopian and inevitable* versus *malleable and hopeful*), and how scientists position themselves in relation to them. The final stage involved elaboration of the rhetorical functions of subject positions (Billig, 1987), providing a comprehensive understanding of how scientists construct the future through their talk to support varied arguments.

*Note*. Scientists often drew on multiple interpretive repertoires, sometimes using different repertoires to serve distinct argumentative functions. This flexibility allowed them to position themselves variably depending on the context, reflecting the multifaceted nature of their engagement with future‐oriented discourse.

## ANALYSIS

We observed that when scientists discuss the future in the context of climate change, they do so within distinct *temporal framings—*discursive constructions of time that organize their talk and support particular arguments. Our analysis identified three key temporal framings in scientists' future talk: *Fixed Futures*, *Delayed Futures*, and *Transformable Futures*. These framings revealed how scientists position themselves within climate discourse, make arguments about the future, and justify responses to climate change, reflecting their constructions on whether and how the future can be influenced.

The degree of fixity or openness in these temporal framings shaped what we term ‘argumentative flexibility’—the range of arguments available to scientists within each framing. At one end of the spectrum was the *Fixed Futures* framing, where the future is presented as a predetermined trajectory towards collapse. This was often characterized by a sense of inevitability and immediacy, as illustrated here:

Extract 1

7M Marine Ecologist

We've kissed it goodbye [1.5°C target]. I can see the feedback loops kicking in. I can see it in the methane, as the permafrost melts […]. I had a dream last night where I could see the flames from my house and the flames were moving towards me. Suffering and death, that's what we all have in our future and I just… unless this problem dissolves like mist and these seemingly insurmountable problems just disappear, I just don't see anything but a whole lot of suffering, a whole lot of chaos, and a whole lot of death.

Moving along the spectrum, the ‘Delayed Futures’ framing presented a negative trajectory, that ‘civilisation could collapse within the next few decades’ (Extract 2, 22 M Ecologist), but it maintained that these outcomes can be delayed through action. The future, while still trending negatively, was seen as having some degree of openness, allowing for the possibility of intervention.

Finally, at the other end of the spectrum was the ‘Transformable Futures’ framing, where the future is presented as flexible and shaped by human actions. This framing opened up discourses that emphasized the potential for change, arguing that the future is not fixed but rather contingent on present actions, e.g:

Extract 3

15M Physics Professor

Some of the rhetoric coming out of some of the groups is too extreme, getting to the point where the rhetoric is almost saying it is just now too late […]. It is never really going to be too late! Yes, the longer we leave it, the worse it is going to be but there will never be a point where […] we might as well give up.

Variability in how the future was framed revealed important insights into how scientists navigate their roles and responsibilities in the face of climate change and construct their vision for transforming the future (see Table 2). Each of these temporal framings drew upon similar repertoires, such as those concerning social and ecological collapse. However, the argumentative functions of how these repertoires were used shifted depending on the temporal framing. For instance, in a fixed future, talk tended towards doomism, that action is pointless because collapse is inevitable. Here actions like civil disobedience were justified more by moral conviction than by the action's efficacy. In contrast, a delayed future allowed for arguments grounded in the efficacy of collective action. Likewise, the transformable future framing opened up discourses regarding the efficacy of collective action, prefiguration, and techno‐solutionism. Given the importance of temporal framing for organizing scientists' talk about the future, below, we structured the results according to the spectrum of temporal framings, from fixed to transformable futures. This ordering is designed to highlight the progression of perspectives on the future within climate discourse, rather than be indicative of the prevalence of each framing.

**TABLE 2:** Temporal framings, interpretative repertoires, and rhetorical functions in scientists' climate discourse.

Temporal frame	Mobilized interpretive repertoires	Scientists' positions and rhetorical functions
Fixed futures (fixed, imminent, and invariable)	Social collapse, ecological collapse, doomism	Prepping and isolation: Collapse and doomist repertoires are mobilized to rationalize survivalist strategies. Scientists adopting this position argued for self‐sufficiency and isolation, seeing society's collapse as inevitable and preparing individually for the worst outcomes. Counterargument of community building: Some speakers pushed back against individualistic responses (e.g. prepping), arguing instead for communitarian resilience and shared anti‐consumerist practices as more ethical and viable alternatives for dealing with collapse. Moral necessity of civil disobedience: As a response to collapse, civil disobedience is justified as a necessary moral response, to do all that one can do, constructing a consistent moral identity to align with one's conscience. Scientists presented these acts as ethical imperatives, aligning with a sense of integrity, but not with any expectation of averting collapse.
Delayed futures (partially fixed but delayable collapse)	Social & ecological collapse, change via collective action, moralized scientist identity	Collective action to delay collapse: Here, scientists highlighted the efficacy of collective efforts, framing protest and civil disobedience as essential strategies to delay collapse. These repertoires depict collapse as delayable through concerted, immediate action. Moralized scientist identity to obligate scientist action: Speakers adopted a more activist stance, drawing on a moral responsibility linked to their professional identity. The scientist identity here becomes intertwined with the idea of acting visibly and ethically in public, pushing for systemic change to protect future generations (*also present in Transformable Futures). Rallying Other Scientists: Through this frame, scientists construct arguments to mobilize their peers, calling for urgent action and framing their activism as both necessary and effective for delaying negative climate outcomes (*also present in Transformable Futures).
Transformable futures (contingent, open, and transformable)	Social collapse, ecological collapse, social change through collective action, prefiguration, techno‐solutionism	Rejecting doomism with measured optimism: Collapse repertoires are used to critique doomist rhetoric, offering a more hopeful yet cautious perspective. Scientists argue that while the future is uncertain, there is still room for meaningful progress, and small victories—like slower emissions growth—are proof of potential positive change. This framing engenders hope and encourages further action rather than resignation. Mobilizing collective action for systemic change: Collapse is framed as a possibility that can still be averted. Scientists argue that human agency, through collective efforts and systemic political change, can shape the future, drawing on discourses of social and ecological collapse as a warning rather than a certainty. Prefigurative Practices and Community Building: Scientists invoke prefigurative practices, advocating for the creation of resilient communities in the present as a solution to the environmental crisis. These discussions draw on hope rather than resignation, positioning present‐day practices as a way of shaping long‐term, sustainable societal change. Technosolutionism: Argument that technology and research are the primary driver of change, focusing on scientific expertise and economic incentives to drive technological solutions to the crisis. Critical perspective on technosolutionism: Warning that overreliance on technology without political and societal change is insufficient to solve the crisis.

### Fixed futures

The Fixed Futures framing was reflected in scientists' mobilization of social and ecological collapse repertoires, representing a viewpoint where the future is perceived as unchangeable and imminent, evoking a sense of doom and inevitability (see e.g. Celermajer et al., 2024; Lamb et al., 2020). Extract 4 illustrates this fixed framing, where the speaker expresses a resigned acceptance of societal collapse:

Extract 4

18F Psychologist

About 10 years ago, I started preparing for the collapse of society […]. It's going to happen […] it's started. There is no evidence to show me that those in power will act […]. I realised that money and the elite will always be prioritised over the needs of the least […]. I've accepted that what is required to save humans is not going to happen […] but because I have children now, I have huge sadness about that […]. But I have accepted that that's going to happen, and it has started happening.

Here, the psychologist frames the future as inevitable, highlighting a pessimistic representation of human inaction and prioritization of elite interests. This sense of inevitable societal collapse is central to the ‘Fixed Futures’ framing, where irreversible collapse is seen as already underway.

In response to this looming future, some scientists presented civil disobedience as a moral obligation. This was exemplified by a marine ecologist, who invoked an assumed societal collapse to justify civil disobedience and the construction of a moral identity:

Extract 5

7M Marine Ecologist

There is a risk of the system coming down on me very heavily […]. Yet I have this obligation as a father and as a grandfather to do whatever I can […]. I have this image of what my grandchildren's life will be like if we don't turn this around. It's a horrific image that I … I do not contemplate very often; I wall it off. […] So, it's a testing—this is the time when my entire life seems to have come to this focus […]. Do I hold back at some level and […] in which case […] I lose self‐respect. […] Am I a hypocrite or am I not? Am I willing to do whatever it takes? […] It's a moral obligation.

Despite recognizing the possibility of severe repercussions, this scientist's rhetoric positions civil disobedience as a necessary and ethical response to the crisis, reflecting recent perspectives (see Capstick et al., 2022; Gardner et al., 2022). His talk highlights the tension between personal safety and moral responsibility, suggesting that failure to act would lead to a loss of self‐respect and integrity. Interestingly, this commitment to civil disobedience is not rooted in its effectiveness. Rather, the speaker frames civil disobedience as an act of moral clarity, with the outcomes left to fate or higher powers:

Extract 6

7M Marine Ecologist

I have to concentrate on the process and leave the result to fate or higher power or God, or whatever you want to call it. I really can't judge the effectiveness of what I do […]. I have to be optimistic day by day when intellectually I'm very pessimistic. […] And I tell myself (laughing) […] that I may not be doing much for my grandchildren but I'm here to save the future for the rats, the worms, and the roaches at least.

Here, the focus shifts from tangible outcomes to the personal integrity of the process itself, underscoring a form of activism driven by ethical duty rather than a calculated expectation of success. Activism within this frame serves as a possible coping mechanism and a way to maintain personal integrity in the face of a bleak future.

Further complicating the response to a fixed future was how scientists talked about the ‘burden of knowledge.’ This concept reflects the ways in which scientists describe the impact of their understanding of potential societal or ecological collapse. One scientist, for example, presented his knowledge as a ‘curse,’ using this discourse to explain the need for distraction and coping strategies:

Extract 7

7M Marine Ecologist

It's…you know, it's a curse. It's a curse to have the understanding I carry around, you know. I've never been a depressed person, but it's depressing to think about, so I distract myself. I go almost no place without a book, alright. I always have a book, a book that will take me to another world—this Umberto Eco, Foucault's Pendulum. A book that will take me out of this depressing world and take me into another world where I know I'm not going to die … and then that's my way of turning off. I need to do this in order to keep functioning, so I've got these mechanisms that will allow me to continue the fight, continue functioning.

In this talk, the scientist frames his actions as part of a broader discourse on managing the burden of scientific knowledge. By portraying his knowledge as a ‘curse,’ he constructs a rationale for employing coping mechanisms, positioning these strategies as essential for continuing his work and engagement with the crisis.

His talk then shifted to considering a longer‐term view of life on Earth to construct an argument about life's resilience:

Extract 8

7M Marine Ecologist

I hope that things don't get so bad that the rats, the roaches, and the worms won't be able to survive, because 100,000 years after human beings are eliminated from the planet, life will … you can't, we won't be able to kill life. Life will bounce back. So, it will be a wonderful, beautiful world again, just we won't be in it and maybe that's all for the best. I grieve about the animals we're taking, the beautiful animals we're taking with us. But there will be other wondrous animals in the future that will evolve, you know. My brother's a geologist and he tells me, he says, ‘10 million years we'll just be this little strata in the rocks and that will be it’.

This positions the scientist as someone who, while deeply concerned about the immediate future, finds a form of solace, albeit tinged with grief, in a longer‐term, geological perspective. However, not all interviewees adopted such a position. Others responded to an envisioned fixed future by employing a discourse of practical action and self‐sufficiency, focussed on preparing for the worst outcomes rather than preventing them:

Extract 9

18F Psychologist

10 years ago, I bought the house that I'm in, and I bought it for this specific purpose […]. We produce our own food […] we grow our own veg and we preserve it, we pickle it […]. I have two daughters, so I'm very aware that when society breaks down, women and girls are the most likely to experience sexual and physical violence […]. One of my jobs will be to teach them how to fucking kill somebody with their bare hands […]. People […] in cities are going to have to come […] looking for the resources that we have, and will my kids be able to protect themselves?

Her talk evokes a Hobbesian view of society, a return to a state of nature characterized by self‐preservation and protectionism. Her presentation of social breakdown functions to justify investment in self‐sufficiency and adopting a protective stance, providing motivation for teaching her daughters self‐defence skills. Additionally, this future is invoked to justify her cultivation of a community that shares her concerns and values:

Extract 10

18F Psychologist

I have identified people who will live in my community, because you can't survive on your own […]. So, I have people who […] would be prepared to live here. So, I need a medical doctor, I have one, I have an architect […]. I have a group of people who believe the same thing as I do.

This is an exclusive notion of community, limited to trusted individuals with specific skills and shared values. It represents a closed community, united by a common concern for survival and a guarded stance against external threats. Others construct community so as to critique isolationist tendencies:

Extract 11

13F Anthropologist

I've been following these deep adaptation groups […] and preppers and survivalists […]. The morality of it all can be put aside when it's about survival […]. You can't really blame them […] if changes are not coming soon enough […]. They just try to prepare alone […] and that's completely unhealthy […]. Okay, fine, you managed to fend off a certain group of people, [but] doesn't it make more sense, and isn't it more moral […] to include as much people as possible who then wouldn't be interested in attacking you […]. I think one of the most moral ways of moving forward is probably community building… Only if we are creating […] communal life which is capable of functioning without this sort of consumerist structures… can we hope to survive.

This discourse challenges the prepper‐survivalist mentality by envisioning a communal life free from consumerist dependencies, and that aims to redirect the future from collapse. The emphasis on inclusive, cooperative communities is framed as both a moral and practical strategy for transformative resilience.

In summary, we see emerging within fixed‐future accounts the tension between individual survivalism and collective resilience. On the one hand, the emphasis on self‐reliance and preparedness reflects a view that the future is fixed and unchangeable, necessitating a shift towards self‐sufficiency and exclusive communities. On the other hand, arguments for moral and ethical action underscores arguments for the necessity of continued activism and ethical responsibility, even when the outcome seems uncertain. These perspectives highlight the complex ways scientists mobilize repertoires of social and ecological collapse to navigate the challenges of a perceived imminent future.

### Delayed futures

The ‘Delayed Futures’ framing reflects talk which presents the future as trending negatively, but that the worst outcomes can be delayed. While the social and ecological collapse repertoires are still present, they are mobilized differently compared to the Fixed Futures framing. Instead of conveying inevitability, they are employed to justify and motivate action, particularly through collective endeavours such as protest. For example, one speaker used these collapse repertoires to argue that although societal collapse may eventually occur, activism can delay it, even if only within their lifetime:

Extract 12

22M Ecologist

Since I've got heavily into climate activism and found out so much more about climate, there's been a very selfish element that's coming into it as well, which is self‐defence […]. Civilisation could collapse within the next few decades and quite simply, this might sound awful to say, but if I can postpone that until after I'm dead, I'll be really happy if I don't have to see it, and so honestly […] there's an element now I'm doing it, you know, to secure for […] my own comfort of my future, in addition to the moral things.

Here, the speaker emphasizes that while collapse is a possibility, collective action is framed as an effective way to postpone it. This temporal framing allows for discourse around the efficacy of collective action, contrasting sharply with the resignation seen in the Fixed Futures framing. The speaker draws on social change discourse, presenting activism as a pragmatic response to the crisis. Moreover, this future framing, by allowing for talk on efficacy, enables the speaker to mobilize the notion of a moralized scientist identity present in the wider discourse (see Gardner et al., 2021; Gardner & Wordley, 2019). This notion posits that scientists' expertise incurs a moral obligation to act visibly in the public interest. The speaker in the following quote uses their identity as an earth scientist to emphasize the importance of visible action, a point also explored in earlier research (Finnerty et al., 2024a):

Extract 13

22M Ecologist

I'm not just any scientist, I'm an earth scientist. I specifically know about what's happening to the planet and […] my knowledge compels me to act […]. I think it also gives me a particular responsibility […] to be visibly doing something because […] I worry that there might be people out there thinking well, if it was really that bad then the scientists would be freaking out. […] I think it's important that scientists are visibly freaking out.

Here, the Delayed Futures framing allows the speaker to justify their activism and call for more engagement from fellow scientists, particularly by framing collective action as a means of delaying catastrophic outcomes. The sense of efficacy within this framing makes the argument for a moralized scientist identity possible.

### Transformable futures

‘Transformable Futures’ represents a temporal framing where the future is spoken of as contingent, open, and still subject to change. While scientists' talk characterized in this way did not ignore the backdrop of ongoing climate and ecological decline, it emphasized the potential for action to shape the trajectory of the future. The following speaker exemplified this framing:

Extract 14

15M Professor Physics

Some of the rhetoric coming out of some of the groups is too extreme, getting to the point where the rhetoric is almost saying it is just now too late […]. It is never really going to be too late! […] Yes, the longer we leave it, the worse it is going to be, but there will never be a point where […] we might as well give up […]. We don't know what the other world could have been—if we had been in a world where we had much more coal use it might have been much worse today than it is. […] Even though emissions are technically still rising they may well be rising more slowly than they might have done, which is a positive even if it is not quite what we wanted […]. It is partly the impact on younger people who might feel despondent, which I don't think is great. And partly the possibility that people do give up and go oh well, there is no point anymore, which I think is certainly not true.

This speaker framed the future as open and transformable to critique ‘extreme rhetoric’ and reject doomism, thereby engendering hope by recognizing incremental progress as valuable and affirming humanity's capacity to avert the worst outcomes of climate change. This position fosters an optimistic, if measured, outlook. Broadly speaking, this open framing permits a broader range of repertoires and arguments to be mobilized, with scientists drawing on wider discourses pertaining to collective action, community building, and technological solutions. Discussions of the future here do not discard social and ecological collapse repertoires but mobilize them differently. They serve as motivation for action rather than as evidence of inevitable doom. Talk of collapse is used to galvanize a sense of urgency, encouraging immediate and diverse responses to the climate crisis. The contingency of the future allows for a wider range of strategies to be discussed and employed, and the focus shifts from resignation to a more proactive engagement with the potential for change.

#### Collective action and prefiguration

One of the key repertoires mobilized within this more open framing was that of social change through collective action. Talk around collective action centred on the argument that widespread mobilization and social movements have the power to alter the trajectory of climate change by creating more sustainable and compassionate systems. Speakers emphasized solidarity and collective affective states (e.g. outrage) as critical tools for change.

Extract 15

5F Sustainability Social Scientist

It just felt so good to be actively doing something and to be in this big, huge groundswell of people who were all really, really angry […]. This is the way forward, it is mass mobilisations, and it is solidarity with other people, and it is people who are angry, and it is not running away from that anger, but leaning into that anger […] that is our only chance really.

Here, collective anger is proposed as a force that can unite individuals and propel them into action. The speaker presents anger not as a destructive emotion but as a source of energy for sustained activism. The speaker further constructs a hopeful path forward through solidarity and mass mobilization, highlighting the importance of community in fostering change.

Another speaker underscored the long‐term, strategic nature of activism, viewing it as an investment in the future. The metaphor of ‘planting seeds’ was used to argue that collective action may not yield immediate results but plays a critical role in shaping the future:

Extract 16

22M Ecologist

With awareness, with alarm‐ringing type actions […] you're planting seeds, and you don't know where and when those seeds are gonna germinate into something […] that's going to be impactful. […]. Activism has put climate on the public agenda in a way that it never was before.

Here, the speaker situates activism as laying the groundwork for future impacts. This long‐term perspective frames collective action as necessary, not only to address present concerns, but also to shape the future through the gradual development of awareness and systemic change.

Prefiguration, a concept emphasizing the embodiment of desired social changes in the present, also emerged as a significant discourse within this temporal framing. This prefigurative approach underlines the necessity of practising the values and systems envisioned for the future in current actions. For example, some speakers articulated the importance of creating and sustaining communities that reflect the values of the future they want to see:

Extract 17

5F Sustainability Social Scientist

I hope that we can all maintain the relationships that we have built with each other and with these communities of people, and I hope that those relationships can just get stronger and stronger and that we create a genuinely regenerative movement… I hope that… we can all help each other and create really strong communities.

This speaker's talk draws on prefigurative discourse to suggest that by building stronger communities now, it is possible to lay the groundwork for a future that can thrive despite the challenges of climate change. This approach serves as a counter‐narrative to social collapse, offering hope and a vision of resilience based on mutual support and cooperation.

Another speaker echoed this sentiment, extending the idea of prefiguration to systemic change:

Extract 18

6F Biologist

A lot of the negative things in the world, all the causes are quite interconnected. So, if we actually created better systems, less individualistic systems, and there's a culture of care about one another, I think that everyone would be happier. Even with all the bad stuff that is inevitably going to happen, I think a better world is possible… They could open so many possibilities to make life better for so many people and that feels great to think about. A human connection, that's what it's all about for me.

Here, the speaker's talk underscores the argument that prefigurative action can create new systems that prioritize care, cooperation, and happiness. This discourse links the openness of the future to collective efforts, reflecting a deeply hopeful perspective on what can be achieved through action and solidarity.

#### Technological innovation as the solution

In contrast to the focus on human‐driven social change, other scientists framed the future as contingent on technological advancements. For them, the primary means of averting environmental catastrophe lies in the development and deployment of innovative technologies. That said, within this view, arguments on the role and sufficiency of technology diverged. Some participants expressed strong faith in the power of science and technology to provide solutions to climate change, suggesting that technological progress offers the most tangible and controllable means of shaping the future, echoing techno‐solutionist discourse. As one speaker noted:

Extract 19

24M Biologist

My hope is that definitely we do have the technology. We do have the brain power to find solutions that can counteract climate change […] but I am aware of literature saying that is not enough, but I think that is more tangible […] because it's in the hands of […] scientists […]. If you want to look at it in a selfish way, it will raise profits and then that will push companies to actively invest in it […]. So, I have more hope on, you know, companies' greed (laughs) than their altruism, to be honest.

Here, the speaker presents technology as a concrete and predictable means of addressing the crisis, with economic incentives and scientific expertise as key drivers of change. Political action, by contrast, is critiqued as less dependable. The speaker further mobilized a defence of technological advancement against critiques that blame science for the current environmental crisis:

Extract 20

24M Biologist

Discussion with my parents would just be […] science screwed everything up. […] Their solution to everything would be to regress […] live life like people used to live a hundred years ago […]. Be at peace with nature, live with nature… not to use much of technology… and… science is the reason why we are facing whatever we are facing. They don't deny climate change, but they also wrongfully accuse science of being the reason for it.

This critique serves to counter the argument that to move forward, we must revert to a pre‐technological era.

Such Panglossian techno‐solutionist discourse was, contrastively, rejected by another speaker who considered it naïve to assume technology will be a panacea for environmental challenges, rather than one tool in the arsenal:

Extract 21

12M Biologist

I'm playing the argument of the technophile […] that eventually we will develop technologies who are going to save the situation without us having to change […] our way of life. So that's where carbon capture and carbon neutralisation come into play […]. That's my job, right? I'm trying to do that, and I can tell you […] unless we also reduce drastically our consumption of fossil fuels […] it's not gonna matter. Like it's taking us so much work to absorb a tenth of the CO2 emission we have emitted. […] I need the politicians to do the rest of the bulk work.

This speaker acknowledges the potential contributions of technological innovations, like carbon capture, while highlighting the need for complementary efforts, such as political action and changes in consumption patterns. This tempered view serves to critique the more extreme position that technology alone can save the future:

Extract 22

12M Biologist

The technological progress since the 70s has been incredible […] but the Keeling curve [graph of atmospheric carbon dioxide accumulation] is the perfect straight curve. […] If the progress we've had in 50 years hasn't been enough to make a dent in it, it's probably not safe to assume it's gonna make a dent in the next 50.

Here, the speaker's reflections on the limitations of past technological advances to reduce carbon emissions is projected into the future and serves as a critique of unbridled optimism about future technologies. Overall, the Transformable Futures framing contrasted sharply with the Fixed Futures framing, where social and ecological collapse were discussed as inevitable. Within a Transformable Futures framing, collapse repertoires were repurposed for different rhetorical purposes, for example, to argue that collapse is possible but not certain, and something that can be averted through collective action, systemic change, and/or technological innovation. This shift underscores how, depending on the perceived openness of the future, collapse discourses move from justifying resignation to motivating proactive and hopeful responses.

## DISCUSSION

The present study explored how environmentally concerned scientists talk about the future in the face of climate change. While there was consensus among scientists on the severity of the ecological crises, there was no uniform agreement on the precise contours of the future. The diversity of epistemic stances highlights the human element within scientific inquiry, and the indeterminacy that characterizes current understanding of complex environmental and social systems. The present interviews revealed a variety of ways in which scientists talk about the future and position themselves in relation to available discourses of collapse (Celermajer et al., 2024; Lamb et al., 2020), techno‐solutionism (Morozov, 2013), and collective action (Berglund & Schmidt, 2020; Fisher, 2024), and argue for responses ranging from individual survivalist strategies to collective action and faith in technological solutions.

### Temporal framings and argumentative flexibility

One key take‐away is that the degree of fixity or openness in how scientists construct the future influences the range of arguments they present when considering potential solutions. Scientists' temporal framings—from *fixed and inevitable* to *contingent and transformable futures*—shaped how they mobilized shared discourses of social and ecological collapse. This variation in temporal framing shaped how scientists positioned solutions to climate change, with more fixed framings leading to a narrower set of potential actions, while more open framings allowed for greater argumentative flexibility. For example, in the Fixed Futures framing, the efficacy of collective action was downplayed or treated with scepticism. The future is presented as beyond the reach of human intervention, with individuals preparing for collapse through personal survival strategies or moral acts of defiance. In contrast, more open temporal framings were associated with talk of both collective action and technological innovation as solutions to the climate crisis. This contrast illustrates how open temporal frames can expand the argumentative flexibility available to scientists in shaping responses to the climate crisis.

### Fixed futures: Resignation and moral action

In the Fixed Futures framing, where collapse is presented as unavoidable, scientists' talk was characterized by a sense of inescapability and *inquiétude* (a feeling of unease or anxiety) (Roux & Lévêque, 2024). Empirical observations, such as methane release from melting permafrost and feedback loops, were invoked to emphasize that critical thresholds are being crossed. This sense of inescapability manifested in two distinct forms of argumentation. First, some scientists mobilized collapse repertoires to rationalize actions that, while proactive in terms of survival, disengage from collective efforts to alter broader societal or environmental trajectories. Echoing apocalyptic discourses (Hoggett, 2011; Lovelock, 2010), some envisioned a grim future of social collapse, a Hobbesian state of nature characterized by self‐preservation and protectionism (Lloyd & Sreedhar, 2022). Prepping behaviours, for instance, were framed as an agentic response of self‐reliance to the anticipated failure of political elites to prevent collapse, in line with other research (Garrett, 2021; Hoggett, 2011; Roux & Lévêque, 2024).

In contrast, collapse repertoires were used by others to justify civil disobedience, not as a means of averting disaster, but as a moral obligation to future generations. In this context, civil disobedience was framed more as a way to maintain personal integrity and ethical consistency (Jennings et al., 2015) than as a realistic attempt to reshape the future. Thus, within the Fixed Futures framing, scientists were able to mobilize both individualistic and collectivistic responses to the impending collapse. However, in contrast to the Delayed and Transformable Futures framings, the Fixed Futures framing confines action to a narrow set of options, each of which is marked by resignation to an inevitable collapse.

### Delayed futures: Activism, ethics, and scientific duty

The Delayed Futures framing characterizes talk which presented the future as trending negatively but still open to intervention. Within this framing, scientists' talk drew upon social and ecological collapse repertoires, similar to those found in the Fixed Futures framing. However, these repertoires were adapted for different rhetorical purposes aimed at delaying collapse. Discussions around collective action, including protest and civil disobedience, were framed as effective strategies for postponing the worst outcomes of the climate crisis, reflecting broader discourses on social change, as seen in movements like Extinction Rebellion (Berglund & Schmidt, 2020; Fisher, 2024). Rather than resigning to an inevitable collapse, this framing supports discourse that foregrounds the efficacy of collective efforts to influence the future's trajectory. By framing the future as somewhat malleable, it also enables the mobilization of a moralized scientist identity (Capstick et al., 2022; Gardner & Wordley, 2019; Rodgers, 2023). In this context, scientists positioned themselves as having a moral duty—not only to understand and communicate the crisis, but to act visibly upon it, using their authority to encourage others to engage in activism (Finnerty et al., 2024a). This sense of responsibility was framed as a broader imperative for scientists to lead by example, using their unique position to influence public perception and policy. The Delayed Futures framing, therefore, opens up space for action‐oriented arguments, where the scientist's role becomes central to delaying negative outcomes through collective and visible activism.

### Transformable futures: Collective action, prefiguration, and technological solutions

Finally, in the Transformable Futures framing, scientists' talk was characterized by the greatest degree of flexibility, allowing the future to be constructed as contingent on present actions. Collapse was not cast as inevitable but as a possibility that can, and must, be averted through human agency. The openness of the future was used to counter doomist discourse (Celermajer et al., 2024; Lamb et al., 2020). Rather than presenting a binary choice between ‘catastrophe or salvation’ (Garrard, 2020), some scientists used this contingency to offer counterfactuals, highlighting the potential for different outcomes based on present interventions. In this context, collapse was reinterpreted not as an unavoidable future but as a call to action, permitting arguments regarding collective action (Ojala, 2023; van Zomeren et al., 2019), prefigurative practices (Berglund & Schmidt, 2020; Yates, 2015), and technological solutions as viable strategies for shaping the future. This optimistic and proactive discourse stood in direct contrast to talk of inevitable social collapse, and, instead, counselled hope as a blueprint for building a better future.

While collective action and prefiguration dominated much of the talk about Transformable Futures, at least one scientist in the sample represented a stronger techno‐solutionist perspective (see Celermajer et al., 2024; Johnston, 2018; Morozov, 2013). This scientist's discourse placed great faith in the power of scientific research and technological advancements for addressing the climate crisis, while simultaneously expressing doubt in individual and government–led solutions. The emphasis was instead on the role of technology in mitigating climate change, a position that reinforced the belief that science and industry, rather than activism, hold the key to future solutions. The techno‐solutionist position might also reflect a particular construction of the scientist identity, one that refrains from activism and places faith in research, technology, and economic systems (Finnerty et al., 2024b). This approach aligns with more traditional notions of scientific detachment, neutrality, and objectivity (Betz, 2013; Merton, 1973).

Other scientists offered critiques of the techno‐solutionist stance, for instance, by pointing to the past 50 years of technological progress, which, despite significant advancements, has not been sufficient to meaningfully reduce global emissions. This more critical stance on technology reflects the broader discourse on the limits of technological innovation, especially where solutions, such as carbon capture and storage or nuclear fusion, remain unproven or face significant ethical and practical challenges (Biermann et al., 2022; Clifford, 2022; Hamilton, 2013; Smith et al., 2023). For example, it has been argued by some that overemphasizing technological ‘myths’ could delay immediate actions, such as efforts to reduce fossil fuel dependency, by fostering a false sense of security (McLaren et al., 2021).

This tension between faith in technology and the critique of its limitations illustrates the breadth of arguments available within the Transformable Futures framing. While some scientists remained hopeful about the potential of technological advancements to drive change, others emphasized the need for a more comprehensive strategy that integrates both technological and socio‐political approaches. This diversity of views underscores how an open‐future framing allowed scientists to entertain a wider array of solutions—both technological and systemic.

### Limitations

Our analysis has several limitations. First, the data primarily came from environmentally concerned scientists in affluent, industrialized countries in the Northern Hemisphere, limiting the global applicability of the study. Given the heightened vulnerability of the Global South to climate hazards (IPCC, 2022), expanding the research beyond a primarily Western or WEIRD (White, Educated, Industrial, Rich, and Democratic) (Henrich et al., 2010; Muthukrishna et al., 2020) sample would enrich understanding of how scientists from different cultural and socio‐political backgrounds discuss the future. Additionally, the sample had a higher‐than‐average proportion of participants engaged in direct activism. While this activist skew provided valuable insights, it may not fully represent the broader scientific community. However, high levels of concern are common among scientists, and a significant minority do engage in direct action (Dablander et al., 2024), suggesting that our findings are still relevant to the broader scientific community. Lastly, the cross‐sectional nature of the study limits our ability to track how scientists' future talk evolves over time. Longitudinal research could offer deeper insights into how this discourse shifts in response to changing conditions and events.

## CONCLUSION: FUTURE TALK AND SCIENTIFIC DISCOURSE

Despite these limitations, this study revealed a diversity of discursive practices within the scientific community, highlighting the complexity of the climate crisis and the varied ways scientists construct the future. It underscores the importance of not just the knowledge scientists contribute to the global conversation about climate change, but also how they articulate potential responses based on their temporal framing of the crisis. The degree to which the future is seen as predetermined or contingent impacts both the urgency and scope of their proposed actions. Recognizing these diverse temporal framings is critical for understanding the range of strategies that may be pursued in response to the climate crisis. Scientists play a critical role in shaping society's understanding and response to the climate crisis. As more scientists lend their expertise to social movements, such as Scientists for Future, and their legitimacy through activism in groups like Scientists for Extinction Rebellion and Scientist Rebellion, it is important to understand how they talk about the future. As we have seen here, the way scientists frame the future sets the boundaries of what actions are seen as possible, viable, or necessary. As trusted figures in public debates, scientists' activism blurs the lines between science, policy, and advocacy. Their engagement in social movements lends expertise and legitimacy to these causes, which in turn may shape climate policies and the public's understanding of what solutions are viable. How scientists frame the future—whether as fixed, delayed, or open to transformation—could potentially influence the focus of climate movements, impacting the urgency, scope, and type of responses pursued. For instance, if doomist or techno‐solutionist discourses were to dominate public communications, they may shape public perceptions of what is necessary and achievable, potentially sidelining social and political action, which many scientists deem essential for addressing the climate crisis. This diversity in scientists' future talk underscores the importance of not just what scientists say, but how they say it. As their voices continue to shape climate discourse, understanding these framings will be crucial for ensuring a range of strategies remain in focus. As society grapples with the escalating climate crisis, recognizing how scientists construct and engage with these futures will be key to shaping effective responses.

## AUTHOR CONTRIBUTIONS

**Samuel Finnerty:** Conceptualization; investigation; methodology; validation; software; formal analysis; writing – review and editing; writing – original draft. **Jared Piazza:** Supervision; writing – review and editing. **Mark Levine:** Conceptualization; methodology; supervision; writing – review and editing; formal analysis; resources.

## CONFLICT OF INTEREST STATEMENT

The authors declare no competing interests.

## ETHICS STATEMENT

We verify that all research reported in this manuscript follows APA ethical standards in the treatment of human participants. The research reported here was approved by the Faculty of Science and Technology Ethics Committee at Lancaster University (Ref: FST‐2022‐0617‐RECR‐3). All participants in this research gave their informed consent prior to their inclusion in the study.

## Supporting information


Data S1.

## Data Availability

The interview transcripts used in this study are not publicly available to protect participant confidentiality. Ethical approval for this research was granted by the Lancaster University Faculty of Science and Technology, and participants provided informed consent with assurances of anonymity. The decision not to make the transcripts publicly available was based on ethical considerations, including the potential for re‐identification of participants despite anonymisation efforts, and the sensitive nature of the topics discussed. Researchers interested in further analysis may contact the corresponding author to discuss the analysis.

## Footnotes

^1^
Techno‐solutionism, the belief that all problems, including social, political, and cultural issues, are best solved by technology (Johnston, 2018; Morozov, 2013) is broadly similar to what was referred to as technological optimism earlier (Lamb et al., 2020; Peeters et al., 2016). For further research on attitudes towards technology in the context of climate change, see (Cologna, Berthold, et al., 2024).
