# Supplementary material for: Climate futures: Scientists' discourses on collapse versus transformation
Source: Br J Soc Psychol. 2024 Dec 14;64(1):e12840. doi: 10.1111/bjso.12840 (PMC11646080; doi:10.1111/bjso.12840)
Supplement: Supplementary file 1 — Data S1. [file BJSO-64-0-s001.docx]

# Supplementary Information

## Appendix A. Supplementary Methods

*Supplementary Table 1 Interview schedule.*

| 1. Tell me about yourself / who are you? |
| --- |
| 1. Would you describe yourself as a scientist / social scientist / academic? /How would you describe your job?   Is this important for how you see yourself?  How would you describe your role in society?  What does success in your role look like? |
| 1. Would you describe yourself as an activist?   Is this important for how you see yourself?  What is the role of the activist in society? |
| 1. Do you see science and activism as compatible? |
| 1. What barriers do you think there are for others like yourself to act?   Have you experienced any of these yourself?  What effect did this have?  How did you overcome it / them? |
| 1. Have you ever engaged in environmental / climate activism? |
| 1. What motivated you to do this initially? |
| 1. What motivates you now? |
| 1. How long have you been engaged in activism? |
| 1. What kinds of action have you participated in? |
| 1. How does it feel to take action? |
| 1. Have you experienced any challenges when taking action? |
| 1. Why have you not engaged in other actions? |
| 1. Where do you draw the line on the actions you take / How do you decide where your limits are?   Do you feel that where you stand on this line might change in future?  If so, how so and why? |
| 1. Is there anything else you do that you feel is effects positive change?   Do you feel that these actions are effective? |
| 1. Which actions are particularly effective? |
| 1. Which actions do you think are ineffective? |
| 1. In which ways do you feel that your work is effective at tackling these issues? |
| 1. Do you know any / other climate activists?   Do you take action with them? |
| 1. Are you a member of any groups?   What has been your experience of being part of this / these groups?  Are these groups important to you? |
| 1. How have your friends and family reacted to your activism? |
| 1. Do any of your friends and family join you in your activism? |
| 1. Some people describe climate change as a moral issue. What do you think about this? |
| 1. For individuals who identified as activists:   Do you feel that how you live your life aligns with your activism?  For individuals who did not identify as activists:  Do you feel that your beliefs or actions regarding climate change align with your everyday life? |
| 1. In general, what is important to you in life?   Does this/do these affect your actions? |
| 1. What is your worry for the future? |
| 1. What is your hope for the future?   How do you think we get there? |

# Data Collection and Recruitment

Natural and social scientists were recruited to the study via an advert included in a survey on scientist activism engagement. An invitation to be interviewed was included at the end of the survey. Seventy-seven participants, out of a final sample of 329, responded to the advert.

Participants were recruited to the survey via opportunity sampling on Twitter and via various scientific societies and were not paid for participation. Recruitment aimed for diversity among natural and social scientists concerned about climate change and who participated or not in climate-related advocacy and activism. Survey responses were collected between February 2022 and October 2022. Interviews were conducted from June 2022 through to December 2022. Twitter was, at the time, a hub for scientific communication and connecting scientists(Stokel-Walker, 2022), and so served as a suitable platform for recruiting scientists. Since its takeover and subsequent change to X many scientists have now left the site(Vidal Valero, 2023), though this occurred after data collection had ceased. Academic societies and environment centres were also targeted, including the Centre for Climate and Social Transformations at Cardiff, the Lund Sustainability Institute, and the Lancaster Environment Centre.

We specifically targeted scientists and social scientists concerned about climate change, whether engaged in activism or not. This focus was crucial for examining activism attitudes and behaviours within the scientific community. Although it excluded unconcerned or indifferent scientists, it aligned with understanding motivations and barriers to activism among those aware of and concerned by the issues. Additionally, both natural and social scientists were recruited to reflect the diverse representation seen in movements like Scientists for Extinction Rebellion and Scientist Rebellion, ensuring a comprehensive view of scientific activism on climate change and representing a wide range of scientific perspectives on environmental activism.

To ensure that as wide a range of viewpoints, experiences, and contexts were captured the following selection strategy was adopted to choose interviewees. In the survey we included a climate advocacy/activism behaviour frequency scale. We calculated descriptive information about advocacy/activism frequency. We divided participants into low, average, and high engagement categories of activism. Fourteen did not engage in any higher risk/higher responsibility activist behaviours (see Supplement for a breakdown of activism behaviours). We aimed to interview 8 - 10 of them (approximately a third of the final interview sample), and a similar number from average and high activism subsets. Participants were chosen at random from each subset using a random number generator. If a participant opted not to be interviewed another participant was randomly selected from these subsets until data collection ceased.

We aimed to conduct a minimum of 25, and maximum of 30, interviews of approximately one hour in length. This was within the resources of the team and ensured a high likelihood of saturation being reached. Saturation, broadly, as noted by Saunders and colleagues can be conceptualized as having been reached on “the basis of the data that have been collected or analysed hitherto, further data collection and/or analysis are unnecessary.”(Saunders et al., 2018) Saturation may be reached when there is enough information to replicate the study, the ability to obtain new information has been attained, and further coding is no longer feasible(Fusch & Ness, 2015). However, a variety of approaches exist in terms of both its conceptualization and application(Saunders et al., 2018). Given these different approaches, and to assure the quality and rigor of our research, we applied the following strategy. Saturation, at the level of data collection, often refers to the number of interviews required until no new information emerges(Saunders et al., 2018). Applying an ‘informational redundancy’ approach(Francis et al., 2010; Guest et al., 2006; Sandelowski, 2008), we determined whether additional interviews were required once the minimum was reached. In contrast to grounded theory approaches, this is a data saturation approach rather than a theoretical saturation approach(Saunders et al., 2018). Two additional interviews were conducted to be certain that interview content did not differ substantially from the previous entries.

**References**

Francis, J. J., Johnston, M., Robertson, C., Glidewell, L., Entwistle, V., Eccles, M. P., & Grimshaw, J. M. (2010). What is an adequate sample size? Operationalising data saturation for theory-based interview studies. *Psychology and Health*, *25*(10), 1229–1245.

Fusch, P., & Ness, L. (2015). *Are we there yet? Data saturation in qualitative research. Qual Rep. 2015; 20 (9): 1408–16*.

Guest, G., Bunce, A., & Johnson, L. (2006). How many interviews are enough? An experiment with data saturation and variability. *Field Methods*, *18*(1), 59–82.

Sandelowski, M. (2008). Theoretical saturation. *The SAGE Encyclopedia of Qualitative Research Methods*, *2*, 875–876.

Saunders, B., Sim, J., Kingstone, T., Baker, S., Waterfield, J., Bartlam, B., Burroughs, H., & Jinks, C. (2018). Saturation in qualitative research: Exploring its conceptualization and operationalization. *Quality & Quantity*, *52*(4), 1893–1907. https://doi.org/10.1007/s11135-017-0574-8

Stokel-Walker, C. (2022). Twitter changed science—What happens now it’s in turmoil? *Nature*, *613*(7942), 19–21. https://doi.org/10.1038/d41586-022-04506-6

Vidal Valero, M. (2023). Thousands of scientists are cutting back on Twitter, seeding angst and uncertainty. *Nature*, *620*(7974), 482–484. https://doi.org/10.1038/d41586-023-02554-0
